# Supplementary material for: Polarized subcellular activation of Rho proteins by specific ROPGEFs drives pollen germination in Arabidopsis thaliana
Source: PLoS Biol. 2025 Apr 21;23(4):e3003139. doi: 10.1371/journal.pbio.3003139 (PMC12043234; doi:10.1371/journal.pbio.3003139)
Supplement: S1 Fig — (A) Phylogenetic tree of the 14 ROPGEFs of Arabidopsis thaliana after alignment of the full-length protein sequence in Jalview, using the integrated MUSCLE alignment tool and calculation of an average distance (BLOSUM62) tree. (B) Presence of transcript or protein for all 14 ROPGEFs in mature pollen, according to the ATHENA – Arabidopsis THaliana Expression Atlas [41]. GEFs are sorted according to the phylogenetic tree. Levels of the transcript are shown in transcripts per kilobase million (TPM) and intensity-based absolute quantification (iBAQ) for protein levels. NA indicates that no transcript or protein was detected in this tissue. (PDF) [file pbio.3003139.s001.pdf]

S1 Fig: Phylogeny of all ROPGEFs of Arabidopsis thaliana and expression levels in mature pollen.

A

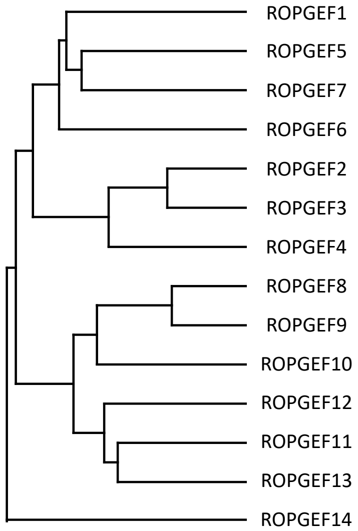

B

Name	Gene ID	Transcript level (TPM)	Protein level (iBAQ)
ROPGEF1	AT4G38430	NA	NA
ROPGEF5	At5G05940	NA	NA
ROPGEF7	At5G02010	NA	NA
ROPGEF6	At3G55660	NA	NA
ROPGEF2	At1G01700	NA	NA
ROPGEF3	At4G00460	NA	NA
ROPGEF4	AT2G45890	NA	NA
ROPGEF8	At3G24620	11.4	24.4
ROPGEF9	At4G13240	11.5	27.1
ROPGEF10	AT5G19560	NA	NA
ROPGEF12	AT1G79860	12.6	26.9
ROPGEF11	AT1G52240	12.4	24.5
ROPGEF13	At3G16130	5.9	NA
ROPGEF14	AT1G31650	NA	17.3

(**A**) Phylogenetic tree of the 14 *ROPGEFs* of Arabidopsis thaliana after alignment of the full-length protein sequence in Jalview, using the integrated MUSCLE alignment tool and calculation of an average distance (BLOSUM62) tree. (**B**) Presence of transcript or protein for all 14 ROPGEFs in mature pollen, according to the ATHENA – Arabidopsis THaliana Expression Atlas [41]. GEFs are sorted according to the phylogenetic tree. Levels of the transcript are shown in transcripts per kilobase million (TPM) and intensity-based absolute quantifications (iBAQ) for protein levels. NA indicates that no transcript or protein was detected in this tissue.
