# Supplementary material for: Polarized subcellular activation of Rho proteins by specific ROPGEFs drives pollen germination in Arabidopsis thaliana
Source: PLoS Biol. 2025 Apr 21;23(4):e3003139. doi: 10.1371/journal.pbio.3003139 (PMC12043234; doi:10.1371/journal.pbio.3003139)
Supplement: S2 Fig — (A) Example images of mCit-GEF13 under the control of a GEF13 promoter fragment in mature pollen grains and pollen tubes grown through a cut pistil. In neither case could any signal be detected. (B) Representative localization of mCit-GEF13 under control of a GEF12 promoter fragment during pollen germination. Timepoint 0 corresponds to the beginning of pollen tube emergence, and arrowheads mark the site of pollen emergence. All scale bars represent 10 µm. (PDF) [file pbio.3003139.s002.pdf]

S2 Fig: mCit-GEF13 is not detectable in pollen and does not accumulate at the pollen germination site.

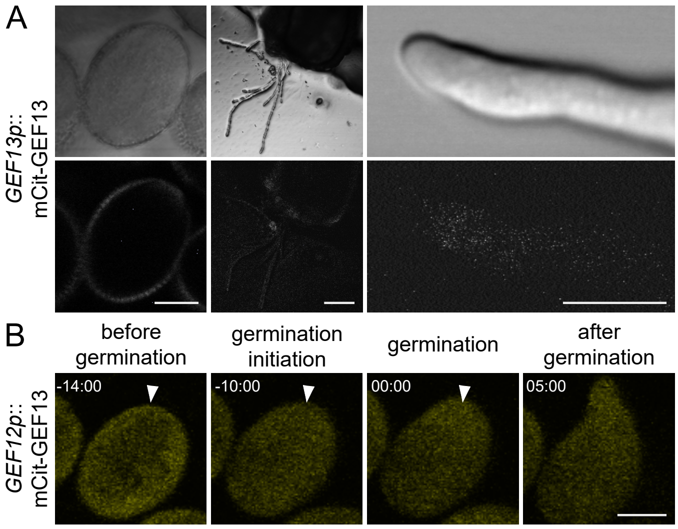

(**A**) Example images of mCit-GEF13 under the control of a *GEF13* promoter fragment in mature pollen grains and pollen tubes grown through a cut pistil. In neither case could any signal be detected. (**B**) Representative localisation of mCit-GEF13 under control of a *GEF12* promoter fragment during pollen germination. Timepoint 0 corresponds to the beginning of pollen tube emergence, and arrowheads mark the site of pollen emergence. All scale bars represent 10µm.
