# Supplementary material for: Polarized subcellular activation of Rho proteins by specific ROPGEFs drives pollen germination in Arabidopsis thaliana
Source: PLoS Biol. 2025 Apr 21;23(4):e3003139. doi: 10.1371/journal.pbio.3003139 (PMC12043234; doi:10.1371/journal.pbio.3003139)
Supplement: S3 Fig — (A–D) GEF8, GEF9, GEF11, and GEF12 genomic structures with the corresponding gRNA sites (scissors) and T-DNA insertion sites (arrowhead) for gef9-t1 (GK-717A10), gef11-t1 (SALK_126725C), and gef12-t1 (SALK_103614). Promoter regions are shown as white boxes, UTRs in cyan, and exons in grey boxes. WT sequence for each gene and corresponding CRISPR/Cas9 deletion line is shown, and the size of CRISPR/Cas9 induced deletions is indicated. The gRNA target sequence is highlighted in color with the PAM in bold. The START and STOP codons are underlined. (E–G) Genomic structures with T-DNA insertion sites (arrowhead) for gef9-t1 (GK-717A10, E), gef11-t1 (SALK_126725C, F), and gef12-t1 (SALK_103614, G) with the location of primers used to test the mRNA presence. The tables show the expected PCR product size with the indicated primer combination for non-spliced templates (genomic) and correctly spliced templates (CDS). The gel images show PCR products of PCRs using the indicated primer combination on cDNA from Arabidopsis flowers of Col-0 in comparison to gef9-t1 (E), gef11-t1 (F), or gef12-t1 (G). (PDF) [file pbio.3003139.s003.pdf]

Fig S3: Genomic structure and mutant allele information of *GEF8, GEF9, GEF11,* and *GEF12*.

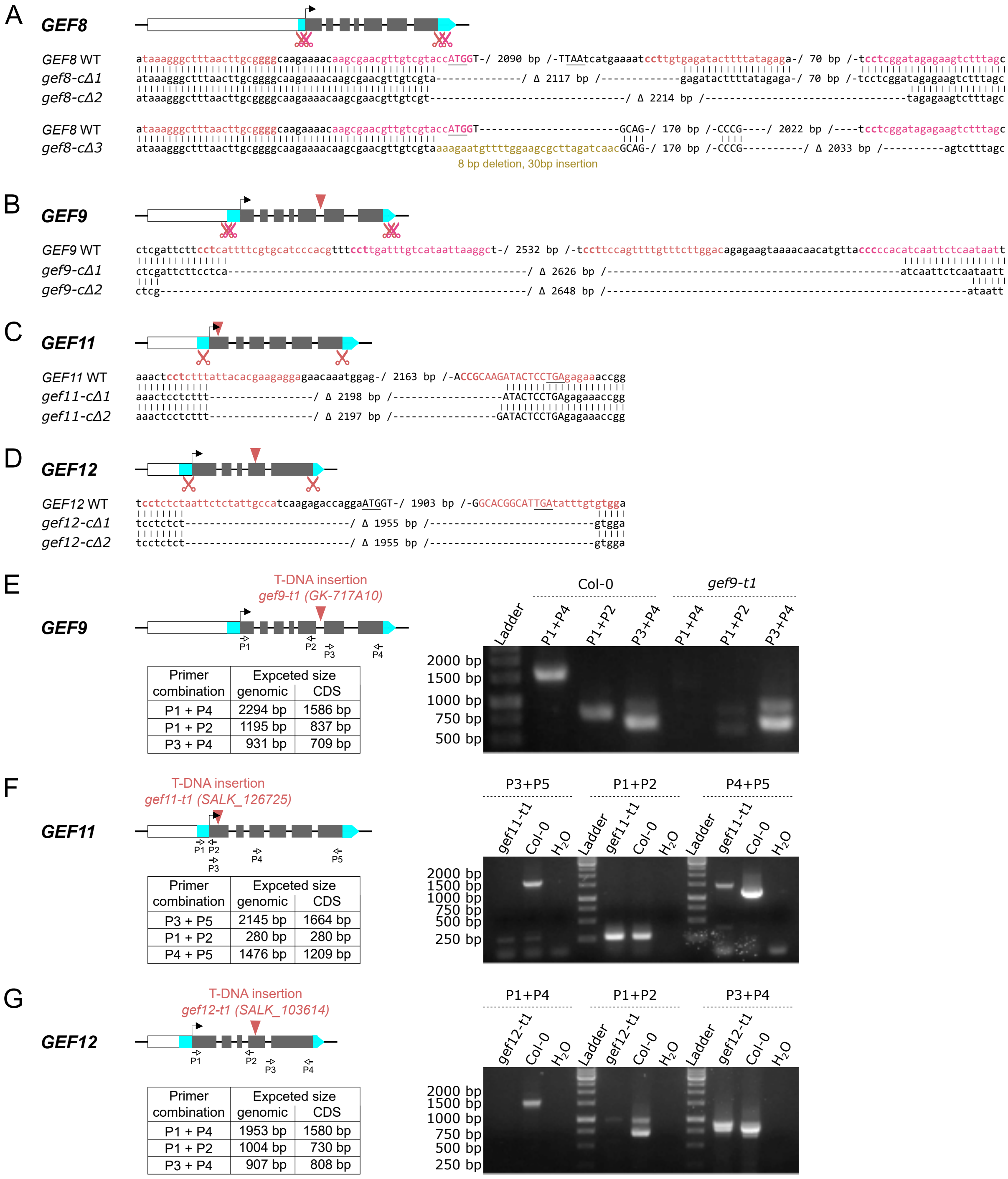

Primer combination	Expceted size genomic	CDS
P1 + P4	2294 bp	1586 bp
P1 + P2	1195 bp	837 bp
P3 + P4	931 bp	709 bp

Primer combination	Expceted size genomic	CDS
P3 + P5	2145 bp	1664 bp
P1 + P2	280 bp	280 bp
P4 + P5	1476 bp	1209 bp

Primer combination	Expceted size genomic	CDS
P1 + P4	1953 bp	1580 bp
P1 + P2	1004 bp	730 bp
P3 + P4	907 bp	808 bp

(**A-D**) *GEF8, GEF9, GEF11,* and *GEF12* genomic structures with the corresponding gRNA sites (scissors) and T-DNA insertion sites (arrowhead) for *gef9-t1* (GK-717A10), *gef11-t1* (SALK_126725C), and *gef12-t1* (SALK_103614). Promoter regions are shown as white boxes, UTRs in cyan, and exons in grey boxes. WT sequence for each gene and corresponding CRISPR/Cas9 deletion line is shown, and the size of CRISPR/Cas9 induced deletions is indicated. The gRNA target sequence is highlighted in colour with the PAM in bold. The START and STOP codons are underlined. (**E - G**) Genomic structures with T-DNA insertion sites (arrowhead) for *gef9-t1* (GK-717A10, E), *gef11-t1* (SALK_126725C, F), and *gef12-t1* (SALK_103614, G) with the location of primers used to test the mRNA presence. The tables show the expected PCR product size with the indicated primer combination for non-spliced templates (genomic) and correctly spliced templates (CDS). The gel images show PCR products of PCRs using the indicated primer combination on cDNA from Arabidopsis flowers of Col-0 in comparison to *gef9-t1* (E), *gef11-t1* (F), or *gef12-t1* (G).
