# Supplementary material for: Polarized subcellular activation of Rho proteins by specific ROPGEFs drives pollen germination in Arabidopsis thaliana
Source: PLoS Biol. 2025 Apr 21;23(4):e3003139. doi: 10.1371/journal.pbio.3003139 (PMC12043234; doi:10.1371/journal.pbio.3003139)
Supplement: S4 Fig — Quantification of mutant allele frequency in F1 generation of reciprocal crosses with Col-0 as female and the indicated mutants as pollen donors (left) or indicated mutants as female and Col-0 as pollen donor (right). The heterozygous allele of each genotype is shown in bold. Asterisks indicate a significant difference in the allele frequency from the expected 50% according to Χ2-test (p < 0.05). For underlying data of all quantification see S1 Data. (PDF) [file pbio.3003139.s004.pdf]

S4 Fig: Reciprocal crosses of GEF mutant lines

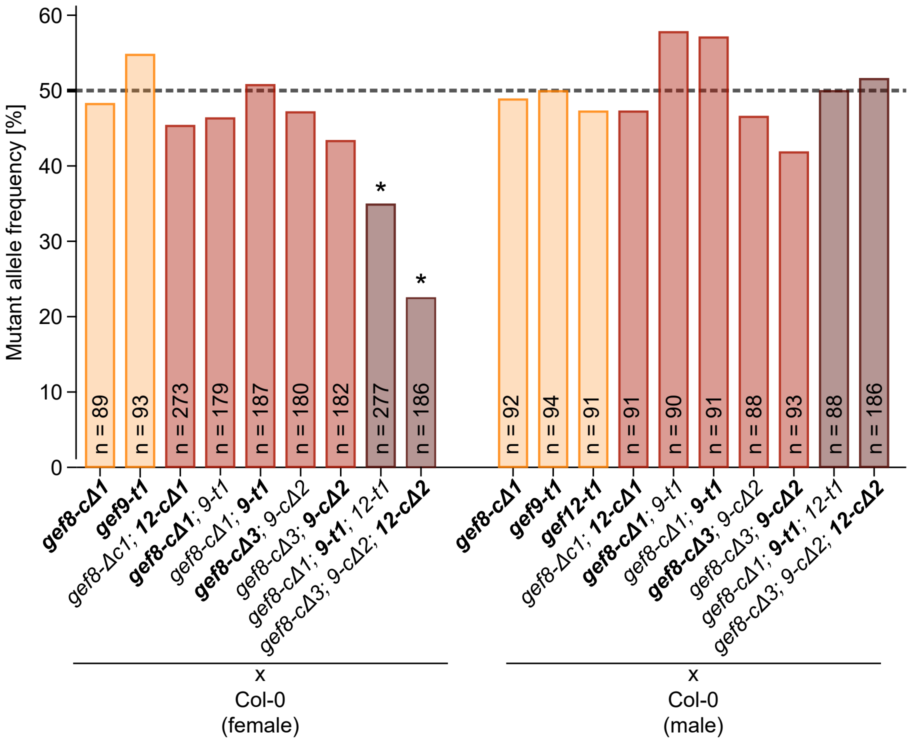

Quantification of mutant allele frequency in F1 generation of reciprocal crosses with Col-0 as female and the indicated mutants as pollen donors (left) or indicated mutants as female and Col-0 as pollen donor (right). The heterozygous allele of each genotype is shown in bold. Asterisks indicate a significant difference in the allele frequency from the expected 50 % according to X^2-test ($p < 0.05$). For underlying data of all quantifications see S1 Data.
