# Supplementary material for: Polarized subcellular activation of Rho proteins by specific ROPGEFs drives pollen germination in Arabidopsis thaliana
Source: PLoS Biol. 2025 Apr 21;23(4):e3003139. doi: 10.1371/journal.pbio.3003139 (PMC12043234; doi:10.1371/journal.pbio.3003139)
Supplement: S5 Fig — (A) Full-length GEF8 and ROP1 protein structures predicted separately by AlphaFold2 (ColabFold v1.5.5) as shown in Fig 3A, and the most likely structure model is shown in different angles [74,75]. The color coding of GEF8 represents the Predicted Local Distance Difference Test (pLDDT) value of the model structure confidence, with blue representing high confidence and orange low confidence. While the conserved PRONE domain has high confidence, the N- and C-terminal regions have very low confidence and contain likely disordered regions. Thus, their structure needs to be taken with caution. (B) pLDDT value per position of all five models. All models have a similar likelihood in the PRONE domain and the terminal regions. (C) The individual predicted structures of GEF8 are color-coded using the pLDDT value, and the terminal amino acids are colored magenta (N) or cyan (C). Below each model, the Predicted aligned error (PAE) data should be considered to assess the domain accuracy of the predicted structures. On the right, the five models are superimposed on each other to highlight the differences. While the PRONE domain largely overlays in all models, the position of the terminal regions and their orientation is different in all models. (PDF) [file pbio.3003139.s005.pdf]

S5 Fig: Prediction of full-length GEF8-ROP1 complex

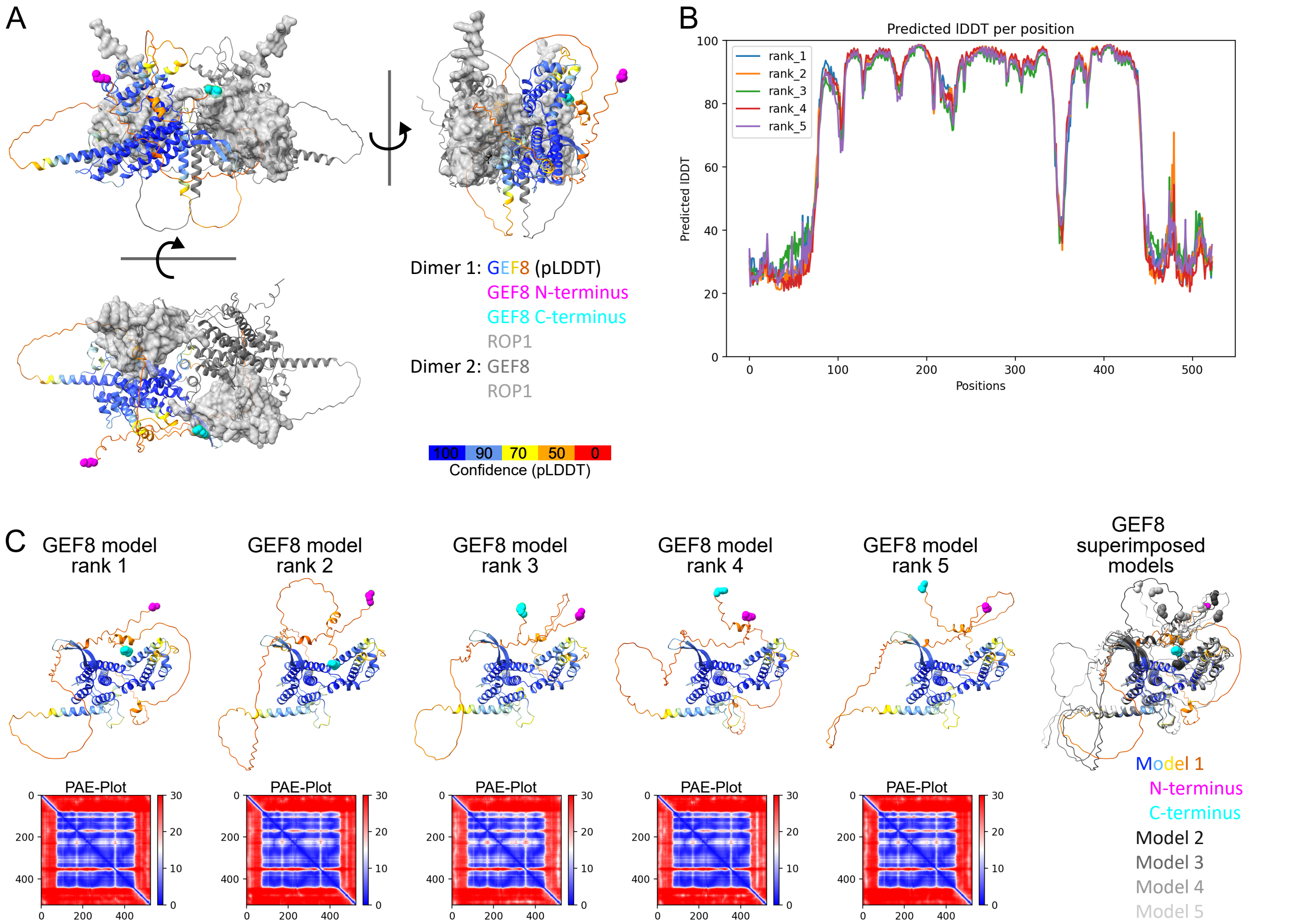

(**A**) Full-length GEF8 and ROP1 protein structures predicted separately by AlphaFold2 (ColabFold v1.5.5) as shown in Fig 3A, and the most likely structure model is shown in different angles [74,75]. The colour coding of GEF8 represents the Predicted Local Distance Difference Test (pLDDT) value of the model structure confidence, with blue representing high confidence and orange low confidence. While the conserved PRONE domain has high confidence, the N- and C-terminal regions have very low confidence and contain likely disordered regions. Thus, their structure needs to be taken with caution. (**B**) pLDDT value per position of all five models. All models have a similar likelihood in the PRONE domain and the terminal regions. (**C**) The individual predicted structures of GEF8 are colour-coded using the pLDDT value, and the terminal amino acids are coloured magenta (N) or cyan (C). Below each model, the Predicted aligned error (PAE) data should be considered to assess the domain accuracy of the predicted structures. On the right, the five models are superimposed on each other to highlight the differences. While the PRONE domain largely overlays in all models, the position of the terminal regions and their orientation is different in all models.
