# Supplementary material for: Polarized subcellular activation of Rho proteins by specific ROPGEFs drives pollen germination in Arabidopsis thaliana
Source: PLoS Biol. 2025 Apr 21;23(4):e3003139. doi: 10.1371/journal.pbio.3003139 (PMC12043234; doi:10.1371/journal.pbio.3003139)
Supplement: S6 Fig — (A–H) Individual relative fluorescence intensity profiles at the pollen germination site of different GEF mutant constructs of the measurements shown and quantified in Fig 3. Thin yellow lines show individual measurements, and thick yellow lines represent the average of all samples. As a reference, the average intensity profiles of GEF8 or GEF9 are shown in black, and that of GEF12 as a dotted line. (A) GEF8p::mCit-GEF8ΔN (n = 10), (B) GEF8p::mCit-GEF8ΔC (n = 13), (C) GEF9p::mCit-GEF9ΔN (n = 16), (D) GEF9p::mCit-GEF9ΔC (n = 14), (E) GEF12p::mCit-GEF12GEF8C (n = 13), (F) GEF12p::mCit-GEF12GEF9C (n = 13), (G) GEF8p::mCit-GEF8S518A (n = 21), (H) GEF8p::mCit-GEF8S518D (n = 13). (I) Representative localization of the GEF8 C-terminus (Gef8p::mCit-GEF8CTerm) during pollen germination. Of the 15 observed germinated pollen, of which 4 could be observed for 15 min before germination, none showed any polarization or protein accumulation. Timepoint 0 corresponds to the beginning of pollen tube emergence, and arrowheads mark the site of pollen emergence. Scale bars represent 10 µm. For underlying data of all quantification see S1 Data. (PDF) [file pbio.3003139.s006.pdf]

S6 Fig: The C-Terminus of GEF8 and GEF9 and its conserved phosphorylation site is required for protein accumulation at the pollen germination site.

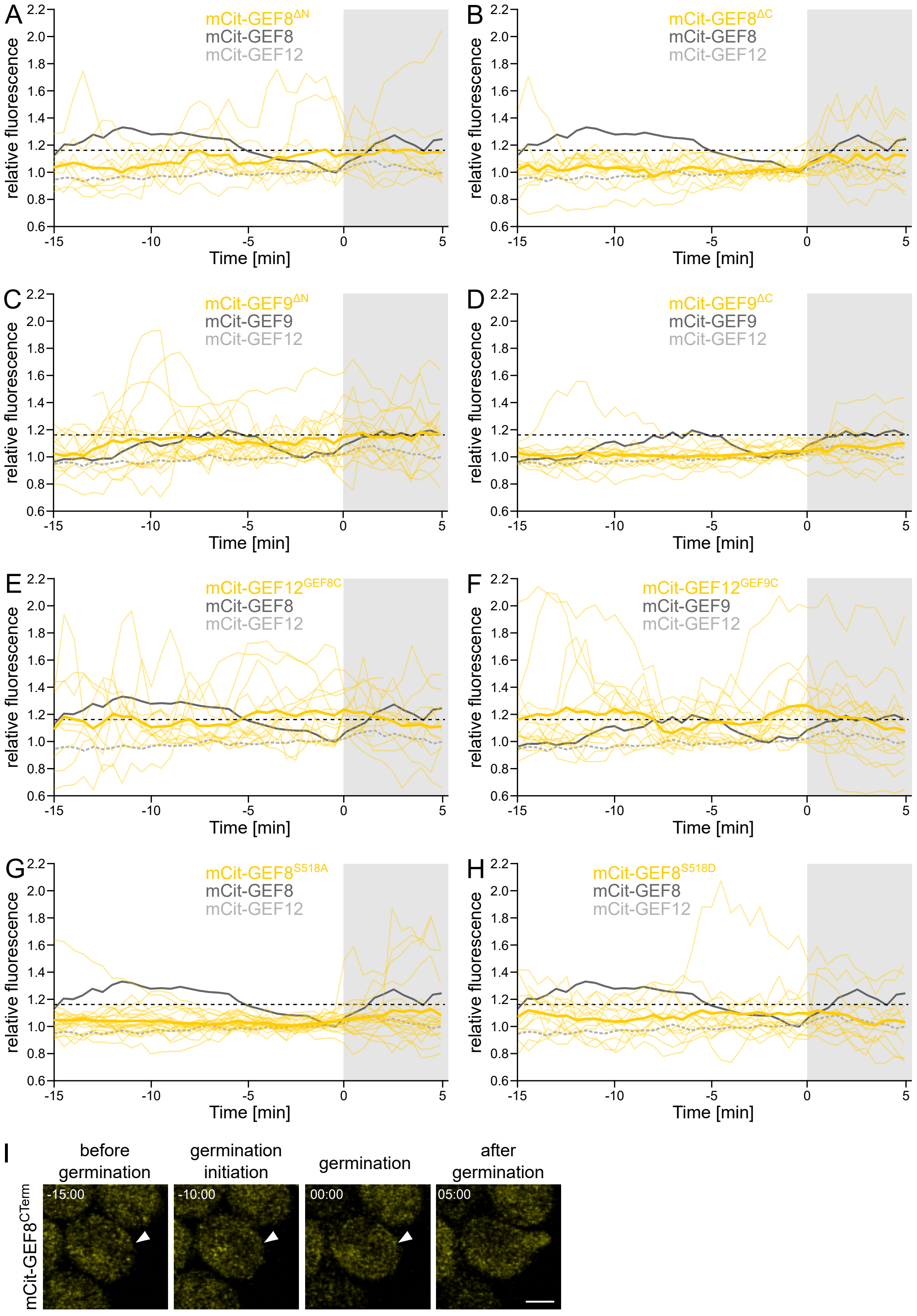

(**A-H**) Individual relative fluorescence intensity profiles at the pollen germination site of different GEF mutant constructs of the measurements shown and quantified in Fig 3. Thin yellow lines show individual measurements, and thick yellow lines represent the average of all samples. As a reference, the average intensity profiles of GEF8 or GEF9 are shown in black, and that of GEF12 as a dotted line. (**A**) GEF8p::mCit-GEF8$^{\Delta N}$ (n=10), (**B**) GEF8p::mCit-GEF8$^{\Delta C}$ (n=13), (**C**) GEF9p::mCit-GEF9$^{\Delta N}$ (n=16), (**D**) GEF9p::mCit-GEF9$^{\Delta C}$ (n=14), (**E**) GEF12p::mCit-GEF12^{GEF8C} (n=13), (**F**) GEF12p::mCit-GEF12^{GEF9C} (n=13), (**G**) GEF8p::mCit-GEF8^{S518A} (n=21), (**H**) GEF8p::mCit-GEF8^{S518D} (n=13). (**I**) Representative localisation of the GEF8 C-terminus (Gef8p::mCit-GEF8^{CTerm}) during pollen germination. Of the 15 observed germinated pollen, of which 4 could be observed for 15 min before germination, none showed any polarisation or protein accumulation. Timepoint 0 corresponds to the beginning of pollen tube emergence, and arrowheads mark the site of pollen emergence. Scale bars represent 10µm. For underlying data of all quantifications see S1 Data.
