# Supplementary material for: Polarized subcellular activation of Rho proteins by specific ROPGEFs drives pollen germination in Arabidopsis thaliana
Source: PLoS Biol. 2025 Apr 21;23(4):e3003139. doi: 10.1371/journal.pbio.3003139 (PMC12043234; doi:10.1371/journal.pbio.3003139)
Supplement: S7 Fig — (A) Individual relative fluorescence intensity profiles at the pollen germination site of Gef12p::CRIB4-mCit in Col-0 (yellow, n = 12) or gef8-cΔ1 (black, n = 12) background, as they are shown and quantified in Fig 4. Thin lines show individual measurements, and thick lines represent the average of all samples. (B, C) Normalized intensity plots of Lat52::RGeco1 in Col-0 (B, n = 13) or gef8-cΔ1 (C, n = 7) background. Black lines represent the normalized RGeco signal intensity. Magenta lines show the significance threshold used to define large Ca2+ elevations. The top graphs correspond to the measurement shown in Fig 4. For underlying data of all quantification see S1 Data. (PDF) [file pbio.3003139.s007.pdf]

S7 Fig: Individual measurements of mCit-CRIB4 and RGeco1 at the pollen germination site.

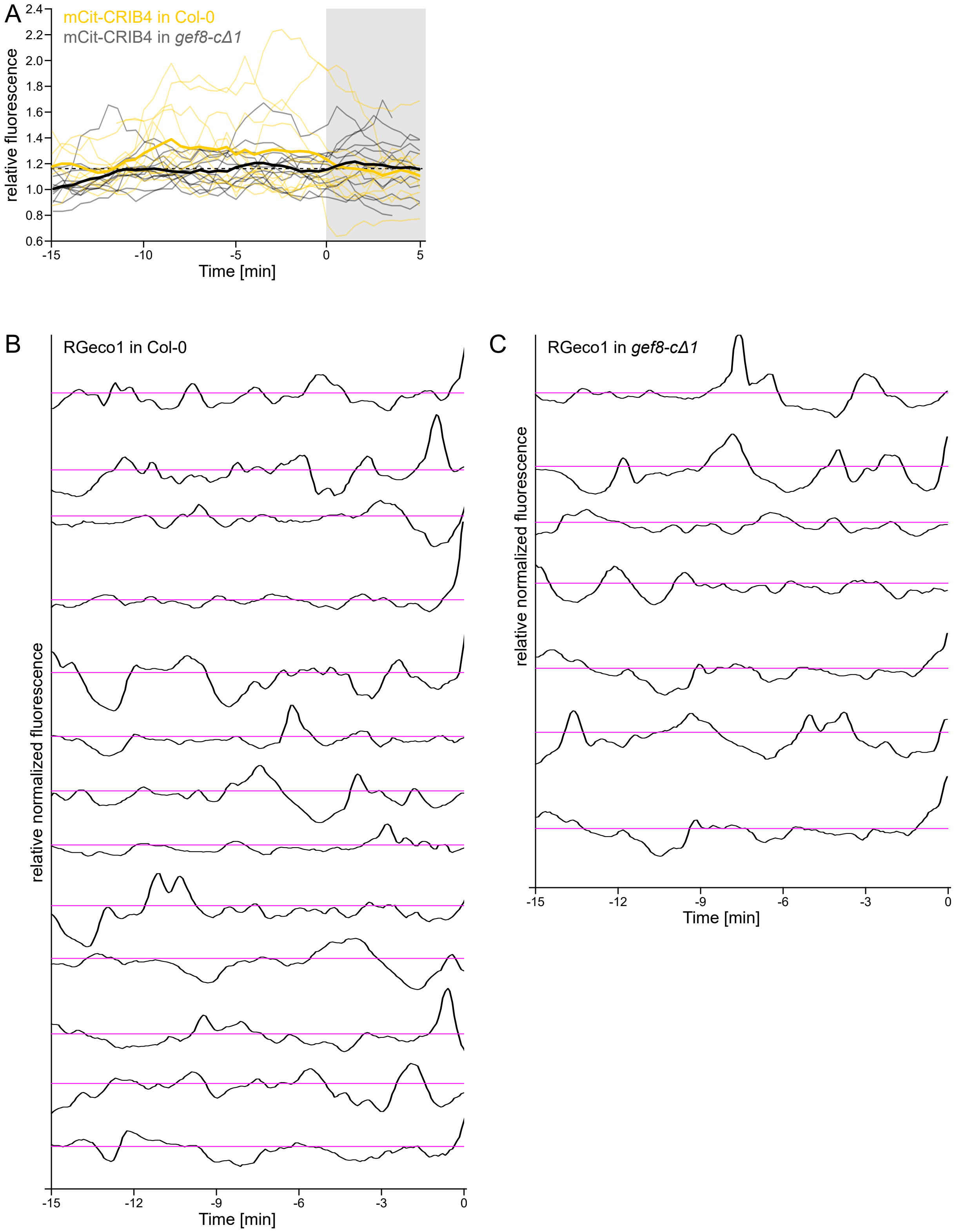

(**A**) Individual relative fluorescence intensity profiles at the pollen germination site of Gef12p::CRIB4-mCit in Col-0 (yellow, n=12) or *gef8-cΔ1* (black, n=12) background, as they are shown and quantified in Fig 4. Thin lines show individual measurements, and thick lines represent the average of all samples. (**B, C**) Normalised intensity plots of Lat52::RGeco1 in Col-0 (B, n=13) or *gef8-cΔ1* (C, n=7) background. Black lines represent the normalised RGeco signal intensity. Magenta lines show the significance threshold used to define large Ca^{2+} elevations. The top graphs correspond to the measurement shown in Fig 4. For underlying data of all quantifications see S1 Data.
