# Supplementary material for: Polarized subcellular activation of Rho proteins by specific ROPGEFs drives pollen germination in Arabidopsis thaliana
Source: PLoS Biol. 2025 Apr 21;23(4):e3003139. doi: 10.1371/journal.pbio.3003139 (PMC12043234; doi:10.1371/journal.pbio.3003139)
Supplement: Attachment — Submitted filename: 143525_1_rebuttal_3073008_sg5559.pdf [file pbio.3003139.s031.pdf]

Revision Plan

Review COMMONS

Manuscript number: RC-2024-02351
Corresponding author(s): Philipp, Denninger

1. General Statements

This Manuscript intends to extend our knowledge of how cells generally polarise and can establish a polar domain that leads to polar growth. Pollen tubes, and more specifically, pollen germination of Arabidopsis thaliana, are a great model for studying this process, as this cell is dormant and unipolar before the beginning of the germination process. Pollen grains must be activated and establish a polar growth domain *de novo*. Despite our extended knowledge about the polarity regulators during the tip growth of pollen tubes, little is known about the players that control germination and the initial establishment of cell polarity. Xiang et al. recently showed that RHO OF PLANTs (ROPs) are redundantly required for pollen germination (Xiang et al., 2023, Plant Physiology, doi: 10.1093/plphys/kiad196). However, it remains unclear how ROPs are initially polarised at the pollen germination site and specifically activated. We tackled this question and found that the known ROP activators, ROP GUANINE NUCLEOTIDE EXCHANGE FACTORS (ROPGEFs), are crucial upstream regulators of ROPs. We show that ROPGEFs are required for polar ROP activation and regulation of downstream Ca2+ signalling and describe the timing of this polarisation event before pollen germination. Moreover, we show that ROPGEFs are generally required for pollen germination and that the involved ROPGEFs are not acting redundantly like it was described for their target ROPs. This demonstrates that ROPGEFs are crucial to implementing specificity in ROP signalling.
Overall, we provide new knowledge about the molecular processes required for the initial steps of pollen germination. Moreover, our findings will help us understand the mechanisms necessary for cell polarisation and polar growth initiation.

2. Description of the planned revisions

We thank all three reviewers for the constructive and well-justified comments and suggestions. All comments will be addressed, and we will implement as many suggestions as possible. We required some time to generate the necessary lines, and we are currently obtaining and analysing more experimental data, as the reviewers suggested. However, we are confident that this new data can dispel potential doubt about our results and strengthen our manuscript in the points raised by the reviewers. Thus, we appreciate the reviewer's comments, as they significantly improve our revised manuscript.

Revision Plan

Response to Reviewer #1:

We agree with Reviewer 1 that a function of ROPGEFs in this process was expected to some degree. However, we want to point out that this manuscript focuses on the requirement of ROPGEFs and especially the spatio-temporal description of ROP signalling polarisation and activation during pollen germination. Moreover, different to the downstream ROPs, we show ROPGEFs do not act strictly redundant, confirming results from root hair initiation and providing additional evidence that multiple signalling pathways are required for pollen germination and that ROPGEFs might be essential for bringing specificity to these signals.

Major comments:

1. Only one GEF11 mutant line, gef11-t1, was analyzed for germination ratio. It is presumptuous to conclude that GEF11 has no function in the pollen germination of Arabidopsis thaliana (line 241- line 242).

After the initial negative results, we did not focus on GEF11 further. Thus, we fully agree that it is presumptuous to make such strong statements about the role of GEF11 during pollen germination. We generated additional gef11 mutant alleles for this revision plan using CRISPR/Cas9 as no other suitable lines were available. Moreover, we now have additional higher-order mutants available to demonstrate the function of GEF11 during pollen germination. These additional lines were generated and confirmed and are growing right now. Thus, we will be able to implement new results addressing this point timely, allowing us to make a more founded statement about the function of GEF11 (see Response to Reviewer #2).

Minor comments:

1. In Figure 2A, pollen germination ratio was not provided for the single mutants gef8-c△3 and gef9-c△2.

This is due to the generation process of the CRISPR/Cas9 alleles. These alleles were generated by a construct mutating both genes simultaneously; thus, these mutants are unavailable as single mutant lines. Instead of separating these alleles by outcrossing, we included additional single mutant alleles for both GEFs with a similar deletion. As all these CRISPR/Cas9 mutants have a complete deletion of the GEF-ORF, we are sure about the loss of the according GEF function. Additional alleles account for possible unspecific effects.

2. In Figure 3D, the subcellular localization of GEF12GEF8C is fuzzy. Better imaging is needed.

We agree that the quality of these images is not ideal due to this specific line having less fluorescent signal. We screened for more lines of this construct and already performed more experiments. We will provide better images for this genotype.

3. In Figure 3E, it is intriguing that both GEF8-S518A and GEF8-S518D are not associated with the PM in germinating pollen grains. Does it mean that phosphorylation at S518 is not relevant to polar distribution of GEF8?

We also find this very intriguing as we did not expect this result. However, we interpret it slightly differently in the way that the S518 site is relevant for GEF polarisation, which might be conferred by RLK interaction. We think both mutant forms alter this potential association with RLKs, thus losing polarisation. We will include more imaging experiments of these constructs and additional lines to strengthen our results. Moreover, we generated lines to study these lines' functionality and complementation capacity, which will be included in a revised manuscript.

4. T-DNA insertion lines, gef11-t1 and gef12-t1, need to be verified by PCRs in Figure S3D.

Thanks for pointing this out. This control should be provided, and we will include the verification in the supplement.

Response to Reviewer #2:

Like Reviewer #2, we are also very intrigued by the biphasic accumulation of GEFs, as this is an entirely novel feature of this process. Like Reviewer #2, we also interpret this as an exploration and establishment phase, which could help us to understand how the pollen germination site is decided in species without aperture-dependent pollen germination.

<u>Major comments:</u>

1. In line 241, the authors conclude that GEF11 has no function in pollen germination. However, it is likely that GEF11 also plays a redundant role as GEF12 does. I recommend the authors check the phenotypes of gef11,gef12 double mutant and gef8,gef9,gef11 triple mutant to confirm that GEF11 has indeed no function. Otherwise, this conclusion should be better rephrased.

This point is well justified and similar to the comment of Reviewer #1. As stated before, we had to generate additional lines for this. We will analyse an additional *gef11* allele, *gef8/gef11* and *gef9/11* double mutants, and *gef9/11/12* triple mutants to address the function of *GEF11* in more detail. The conclusions of the original manuscript will, of course, be adjusted according to the new results.

2. Although GEF12 is in the cytosol, the strong pollen germination defects in gef8,gef9,gef12 triple mutants do indicate a critical role of GEF12. Is it possible that GEFs could function in the cytosol? The authors can test this possibility by examining the rescuing ability of several constructs that express, for example, GEF12, GEF12(+GEF8C), GEF8(SA), or GEF8(SD) in gef8. The authors may not perform all of these rescue experiments, but some of the mentioned lines are already in hands. They could readily check the phenotypes.

We thank the Reviewer for this great point. This information is crucial to discriminate the function of the individual GEFs. We have generated new lines expressing some of the mentioned constructs in the gef8 background to address this. We now have lines that complement *gef8* with GEF12, $GEF12^{GEF8C}$, $GEF8^{S518A}$, $GEF8^{S518D}$, and $GEF8^{\Delta C}$. We are currently performing experiments which determine the functionality of these constructs, which will allow us to make more conclusive statements about the function of GEFs in the cytosol and how important the PRONE domain alone, or the membrane attachment of GEFs, is for their function.

3. The authors conclude that the C-terminus of GEF8 and GEF9 is necessary and sufficient for membrane localization because GEF8/9C can target GEF12 PRONE domain to the membrane. It is intriguing whether the C-terminus alone could confer membrane targeting ability. Currently, it is not fully understood how GEFs localize to the membrane. Examining the localization of GEF8/9C itself would help clarify this and improve our understanding of GEF regulation. Alternatively, the authors may discuss evidence that supports or disagrees with this possibility.

This is a good suggestion by the reviewer and indeed intriguing if the C-Terminus alone could confer membrane attachment. Meanwhile, we obtained plants expressing such constructs, showing that the C-terminus alone is insufficient for membrane attachment. This is not surprising, as these domains are largely disordered, and we suspect that the context of an adjacent PRONE domain is required to carry out this function. We will include our new results in the revised manuscript.

Minor comments:

1. The N- and C-terminus of GEF8 are predicted to inhibit complex formation. How is the prediction performed? Do the authors use monomer prediction or multimer prediction? Alphafold2 has a low accuracy in predicting non-conserved regions. How confident are the predicted inhibitory contacts?

We used multimer-prediction of Alphafold2 for the shown structures. However, we fully agree that the predicted structures of Alphafold have low accuracy in that regard, especially for disordered domains like this. We will provide confidence models and predicted aligned error (PAE) plots for this structure. Additionally, we will put our conclusions in a better perspective of these structure confidences and tone down our interpretations of this section.

2. Localization of ROPs and calcium reporter in Figure 4 appears to be variable. It would help clarify the specific effects on each reporter if the authors present these data more quantitatively.

We agree with the reviewer that some of the observations are variable. We will provide the data more quantitatively, including overviews of which percentage we observed the described phenomena and a more quantitative analysis of the strength and timing of signal accumulation (see also Response to Reviewer #3).

Response to Reviewer #3:

Major points:

1. One of my major points is that the manuscript is now mainly based on the observations of individual pollen grains. These are then subjected to well-performed image analysis approaches but still represent somewhat anecdotal evidence (Fig 1A, B, Fig 3C-E, etc). The analysis and (numerical) presentation of a more robust data sample (which I presume the authors have acquired) would strengthen the ms considerably. This goes beyond the Figs - e.g. in l. 164-165 authors state rather vaguely, "we observed that mCit-GEF8 and mCit-GEF9 accumulated at a defined region in the cell periphery, which strongly correlated with the future germination site." Here, I would appreciate the data showing the actual correlation, if every germinated pollen grain displays GEF8/9 accumulation, whether there is a population of pollen grains showing the GEF8/9 transient but not germinating, etc...

We very much appreciate the reviewer's comment, as this version of the manuscript indeed seems like we made our conclusions based on observations made from individual pollen. However, this is not the case. As the reviewer suspected, more data is available but not included in the manuscript. We have multiple observations for each of the shown constructs and only show a representative one. Furthermore, we imaged more pollen germination events of lines that showed variability and included additional lines for some constructs. We will provide a more quantitative analysis of the results to better represent the variability of the individual constructs, and we will adjust the manuscript accordingly (see comment 2).

2. Where the authors analyse multiple cells, we are still missing some info - e.g. it is not stated what the error bars in Fig 1C, D represents (SD, SEM, CI?), size of the sample, etc. In any case, it is evident that there is quite substantial variability in the data, which is understandable. Maybe the authors can plot the individual profile lines along the average? Plus, GEF9 seem to have the maximum pre-germination localisation at -5 min rather than -9 min.

We agree with the Reviewer that information is missing or not obviously stated. We will correct this for the revised manuscript. Moreover, we agree that the suggested way of showing the data would provide more information and allow a better representation of the results and their variability. This can be seen in the reviewer's interpretation of the results of GEF9. In this case, we see some variability in the timing of GEF9 accumulation, leading to the peak maximum shift. In a revised manuscript, we will, as suggested, show the data as individual lines, providing a better representation of the data. Moreover, we will include such representations for other used constructs to provide a general, more quantitative data analysis (see comment 1).

3. I know it is very challenging, but the ms would be much stronger with the in vivo imaging of pollen germination on stigmatic papillae (i) GEF8/9 in wt, (ii) gef8/9 double mutant. This would bring crucial data about the role of the GEF polar domain and its functional relation to pollination.

This would indeed be great to see. We put an effort into establishing such in vivo imaging experiments with our fluorescent markers. However, we cannot image these events in an *in vivo* setup (at least with our resources). This has two reasons: 1. The events are very fast and limited to a small region at the pollen-papilla contact side, which we have issues resolving optically and timely. 2. The used marker lines only have a low fluorescent level due to the native promoter, and stronger expression would lead to overexpression artefacts. *In vitro*, it is difficult to see the observed signal accumulation. In the in vivo situation, we are facing additional diffraction of the papilla cells, which would make the observation of GEF accumulation impossible with our microscopes.

4. The phylogeny presented in Fig S1 is only rudimental and not very interesting. Given the author's results, I would love to see if GEF8/9 orthologs also exist in species with defined pollen apertures (where establishing a dynamic site makes little sense). The authors touch on this (L409-411), but it would deserve better analysis and discussion.

We agree with the reviewer that studying GEF function/accumulation in species with aperture-dependent germination would be interesting. However, we can not conclude functional orthologs in other species based on phylogeny. Such phylogenetic analyses were done, for example, by Kim et al. (BMC Plant Biology, 2020, doi: 10.1186/s12870-020-2298-5). The issue is that all Arabidopsis pollen-expressed GEFs form a closed phylogenetic group

without allowing the interpretation of which rice homolog is the functional ortholog of the respective Arabidopsis GEF (this is the same for maize). Thus, such phylogenetic analyses are not conclusive, and they would require experimental data to prove orthology. However, we agree that this point can be interpreted and discussed better, and we will include this in the revised manuscript.

5. I am not entirely convinced by the authors' interpretation of rather strange S518 mutation data. Could S518A mutation affect overall GEF8 structure/stability?

We were also suspicious about these results, as they were unexpected (see also Response to Reviewer #1). To confirm these results, we made additional lines for these constructs, double-checked that the constructs were correct and made more observations for both $GEF8^{S18A}$ and $GEF8^{S18D}$. Additionally, we started investigating the functionality of these constructs and have this data available timely. Preliminary results suggest that the constructs are partial to fully functional compared to the WT GEF8, arguing against these mutations' effect on structure or stability. We will include more data for these constructs in a revised manuscript to allow a more conclusive interpretation of these unexpected observations.

6. Although the authors cannot observe the localisation of ROPs in the plasma membrane, they see the apparent accumulation of active ROP marker CRIB4 there - implying that ROPs must localise to the pollen PM at the germination site. This discrepancy should be solved or at least discussed more.

The reviewer is correct in that we cannot observe ROP accumulation but rather the accumulation of ROP activity (as seen by CRIB4). This is in line with the observation made by Xiang et al. (2023, Plant Physiology, doi: 10.1093/plphys/kiad196), which also cannot find ROP accumulation. We are convinced that ROPs are present at the plasma membrane of the pollen germination site, but no accumulation is observable. We believe this is due to a high mobility of ROPs and that no accumulation is required, as only a few ROPs are sufficient to activate downstream signals. We will discuss these results in more detail in a revised manuscript to better explain the observed discrepancy.

7. Given that calcium oscillates very rapidly in pollen and pollen tubes (with frequency ~6-20s), the profound, long-term changes in calcium levels reported by the authors can hardly be referred to as oscillations. The phenomenon observed should again be analysed using a bigger sample.

We agree that the terminology is not good, as it suggests similarities to the oscillations found in pollen tubes. Thus, we will change the revised manuscript and refer to the changes in Ca^{2+} levels as “elevations”. Moreover, we will provide a more quantitative analysis and a bigger sample size, as stated in Response to Reviewer #2.

Minor points:

1. In Fig 1F, GEF12 also seems to be polarly localised to the future site.

The chosen sample is not ideal, as it looks like GEF12 would also slightly accumulate. However, as seen in the quantification of this cell, GEF12 does not significantly accumulate at the pollen germination site, and we never observed any accumulation of GEF12 that is comparable to GEF8 or GEF9. We will include another sample of this colocalisation in the revised manuscript to avoid misinterpretation of the data.

2. It is difficult to make any assumptions based on the AlphaFold2 predictions without showing their confidence assessments (e.g., PAE plots). The authors state this themselves in the discussion (L. 447-449).

As the Response to Reviewer #2 stated, we will include structures with confidence values and PAE plots in the supplement. We additionally tone down our interpretation of these structure predictions to make clear that these structures should be interpreted carefully.

3. On one hand the authors repeatedly state that pollen GEFs do act in a redundant manner (and provide some evidence for it), on the other hand the absence of an in vivo phenotype for single and double knockout lines and only mild phenotype for a triple ko line does suggest a level of redundancy. This should be rephrased.

We agree that this is not clearly phrased. In a revised version, we will change the manuscript to indicate which type and level of redundancy are described. We will discriminate between genetic redundancy, as seen in the mild in vivo effects, and non-redundant molecular function, as observed by protein localisation.

3. Description of the revisions that have already been incorporated in the transferred manuscript

We have not yet made any changes to the transferred manuscript, as we were obtaining additional new lines and, thus, new experimental data. We are currently obtaining and analysing this data. The expected and required changes are substantial, making it useless to implement them without the pending results. The manuscript will be changed according to the results obtained as soon as this data is available.

4. Description of analyses that authors prefer not to carry out

1. Reviewer #1 states that the role of PRKs in GEF accumulation should be studied. We fully agree that this would be valuable, and we already thought of this and suspected PRKs as crucial activators of ROPGEFs in this process. However, the genetic redundancy of PRKs makes this very difficult. As seen in Chang et al. (2013, Molecular Plant, doi: 10.1093/mp/sss103), a prk1/prk2/prk5 triple mutant has altered pollen tubes but no changed pollen germination rate. We suspect at least a quintuple or higher order mutant would be required to obtain a substantial pollen germination defect. As changes in GEF accumulation would only be expected in lines with substantially altered germination efficiency, such experiments and the generation of the required higher-order mutants would exceed the time and scope of this revision.

2. Reviewer #3 suggests in vivo imaging of GEF8/9 marker lines, which we agree would be interesting data. However, due to technical limitations that we discussed in detail in the response to the reviewer section, we cannot provide this data within the scope of this revision. Furthermore, the presented results already provide a novel mechanism that explains an additional step of pollen germination, which we think would not be further improved by *in vivo* imaging.

Revision Plan

Review COMMONS

Original Reviews:

Reviewer #1 (Evidence, reproducibility and clarity (Required)):

In the manuscript, the Denninger group reported the identification of ROPGEF8/9 as the key ROPGEFs for ROP activation during pollen germination, a process of polarity establishment. By examining the subcellular localization of pollen-expressed/enriched GEFs using their own promoter and fluorescence protein fusions, the authors convincingly showed the spatiotemporal distribution of GEF8 and GEF9 during pollen germination. By characterizing pollen germination of gef mutants, the authors demonstrated that GEF8 and GEF9 are critical for the process, with GEF12 playing a redundant role likely as a compensation response. The authors further showed that C-termini of GEF8/9, previously demonstrated as an inhibitory domain for GDP-GTP exchange, was critical for the polar distribution. The C-termini of GEFs interact with PRK. The authors reported that the phosphorylation of GEFs at C-termini was critical for their polar distribution. By examining the dynamic localization of active ROP biosensor CRIBRIC4, the authors demonstrated that GEF8/9 were critical for polar distribution of active ROPs at future germination sites. By introducing a calcium biosensor, the authors showed that calcium gradient, a key downstream process of ROP signaling, was compromised by functional loss of GEF8/9 during pollen germination.

major comments

4. Only one GEF11 mutant line, gef11-t1, was analyzed for germination ratio. It is presumptuous to conclude that GEF11 has no function in the pollen germination of Arabidopsis thaliana (line 241-line 242).

minor comments

1. In Figure 2A, pollen germination ratio was not provided for the single mutants gef8-c△3 and gef9-c△2.
5. In Figure 3D, the subcellular localization of GEF12GEF8C is fuzzy. Better imaging is needed.
6. In Figure 3E, it is intriguing that both GEF8-S518A and GEF8-S518D are not associated with the PM in germinating pollen grains. Does it mean that phosphorylation at S518 is not relevant to polar distribution of GEF8?
7. T-DNA insertion lines, gef11-t1 and gef12-t1, need to be verified by PCRs in Figure S3D.

Significance (Required)):

The identification of ROPGEF8/9 as the key ROPGEFs for ROP activation during pollen germination is a step forward in understanding ROP signaling. Useful but not unexpected. Pollen germination is a process of polarity establishment, similar to root hair initiation. Compared to pollen tube growth and root hair growth, processes of polarity maintenance, the role of ROP signaling was less clear. Recently, Xiang et al. (2023, Plant Physiol) reported an essential role of ROP1/3/5 and their downstream components BDR8/9 in pollen germination. Consistently with polar ROP activation, Ca2+ and post-Golgi secretion were polar. The current work is one step ahead, showing GEF8/9 as the upstream GEFs for this process, comparable to GEF3/4 during root hair initiation (Denninger et al., 2019, Curr Biol). The identification of the C-terminal phosphorylate site in GEF8/9 is informative. It was reported previously that PRKs interact with the C-termini of GEFs to release their auto-inhibition (Gu et al., 2006, Plant Cell; Zhang and McCormick, et al., 2007, Proc Nat Acad Sci USA, Zhao et al., 2013, J Exp Bot) and PRKs were reported to phosphorylate GEF (Chang et al., 2013, Mol Plant). Thus, results reported in the current work indicate that phosphorylation of GEFs likely by PRKs is a critical step for the establishment of polarity domain for pollen germination. From this perspective, it would be more mechanistically sound to investigate the role of PRKs in spatiotemporal polarization of GEFs during pollen germination.Researchers working on cell signaling and cell morphogenesis in plants will be interested.

Revision Plan

My lab works on cell morphogenesis and ROP signaling. This manuscript exactly falls within the expertise of my field.

Revision Plan

Reviewer #2 (Evidence, reproducibility and clarity (Required)):

In this study, Bouatta et al. report the function of RopGEFs in pollen germination. The authors analyzed all of the five RopGEFs, namely RopGEF8/9/11/12/13, that have been shown to be expressed in mature pollen tubes, and found that only GEF8/9/11/12 are detectable. In addition, GEF8 and GEF9 localize to germination sites, while GEF11 and GEF12 are cytosolic. Through a series of phenotype analyses and live-cell imaging, the authors show that GEF8, GEF9, and GEF12 are required for pollen germination while GEF11 is not. The authors also provide evidence that GEF8 and GEF9 are targeted to the membrane via the C-terminus, where they activate ROPs and calcium signaling.

Major comments:

1. In line 241, the authors conclude that GEF11 has no function in pollen germination. However, it is likely that GEF11 also plays a redundant role as GEF12 does. I recommend the authors check the phenotypes of gef11,gef12 double mutant and gef8,gef9,gef11 triple mutant to confirm that GEF11 has indeed no function. Otherwise, this conclusion should be better rephrased.
2. Although GEF12 is in the cytosol, the strong pollen germination defects in gef8,gef9,gef12 triple mutants do indicate a critical role of GEF12. Is it possible that GEFs could function in the cytosol? The authors can test this possibility by examining the rescuing ability of several constructs that express, for example, GEF12, GEF12(+GEF8C), GEF8(SA), or GEF8(SD) in gef8. The authors may not perform all of these rescue experiments, but some of the mentioned lines are already in hands. They could readily check the phenotypes.
3. The authors conclude that the C-terminus of GEF8 and GEF9 is necessary and sufficient for membrane localization because GEF8/9C can target GEF12 PRONE domain to the membrane. It is intriguing whether the C-terminus alone could confer membrane targeting ability. Currently, it is not fully understood how GEFs localize to the membrane. Examining the localization of GEF8/9C itself would help clarify this and improve our understanding of GEF regulation. Alternatively, the authors may discuss evidence that supports or disagrees with this possibility.

Minor comments:

1. The N- and C-terminus of GEF8 are predicted to inhibit complex formation. How is the prediction performed? Do the authors use monomer prediction or multimer prediction? Alphafold2 has a low accuracy in predicting non-conserved regions. How confident are the predicted inhibitory contacts?
2. Localization of ROPs and calcium reporter in Figure 4 appears to be variable. It would help clarify the specific effects on each reporter if the authors present these data more quantitatively.

Advance: ROP GTPases and RopGEFs are critical regulators of cell polarity, but how they initiate polarity remains unclear. This study uses pollen germination as a model to address this question. It systematically analyzed all pollen-specific GEFs and found that GEF8 and GEF9 are critical regulators of pollen germination and polarity initiation. Importantly, GEF8 and GEF9 undergo biphasic accumulation, suggesting polarity is established through a transient exploration phase. This study provides a comprehensive view of the functions of GEFs in polarity initiation, which will be of interest not only to readers who work on pollen germination and growth but also to those who study cell polarity and morphogenesis in general. In my view, the most novel part of this study is that GEFs play overlapping but non-identical roles in polarity establishment and undergo transient accumulation during the polarity initiation process.

Limitations: This study shows that GEFs use the C-terminus for membrane targeting and GEFs can activate ROPs and calcium signaling during pollen germination. These mechanisms could be largely inferred from previous studies in mature pollen tubes or others. Advancements in the regulation of GEF such as how the C-terminus mediates GEF localization, e.g. whether through direct interaction with the PRONE domain in a phosphorylation-dependent manner, would further increase the novelty of this work.

Revision Plan

Reviewer #3 (Evidence, reproducibility and clarity (Required)):

This manuscript investigates the role of PRONE ROP GEFS in germinating Arabidopsis pollen. Given that the molecular mechanisms underlying cellular polarisation in pollen germinating pollen grains are still largely unknown (as opposed to the tip growth of elongating pollen tubes), this manuscript deals with an important topic. Moreover, it builds on the excellent previous research from the lead author, which uncovered ROP GEFs as principal polarisation players during root hair initiation. Here, the authors found that out of five pollen-expressed GEFS, two (GEF8 and 9) mark a future germination site with remarkable spatiotemporal dynamics. Using the genetic tools, GEF8 and 9 were shown to be important for pollen germination in vitro and participate in germination in vivo. Generally, this is an exciting topic, and I quite enjoyed reading the manuscript. However, there are several aspects of the work, which - when addressed - would significantly improve the overall message presented by the authors.

Major points

1. One of my major points is that the manuscript is now mainly based on the observations of individual pollen grains. These are then subjected to well-performed image analysis approaches but still represent somewhat anecdotal evidence (Fig 1A, B, Fig 3C-E, etc). The analysis and (numerical) presentation of a more robust data sample (which I presume the authors have acquired) would strengthen the ms considerably. This goes beyond the Figs - e.g. in l. 164-165 authors state rather vaguely, "we observed that mCit-GEF8 and mCit-GEF9 accumulated at a defined region in the cell periphery, which strongly correlated with the future germination site." Here, I would appreciate the data showing the actual correlation, if every germinated pollen grain displays GEF8/9 accumulation, whether there is a population of pollen grains showing the GEF8/9 transient but not germinating, etc...
2. Where the authors analyse multiple cells, we are still missing some info - e.g. it is not stated what the error bars in Fig 1C, D represents (SD, SEM, CI?), size of the sample, etc. In any case, it is evident that there is quite substantial variability in the data, which is understandable. Maybe the authors can plot the individual profile lines along the average? Plus, GEF9 seem to have the maximum pre-germination localisation at -5 min rather than -9 min.
3. I know it is very challenging, but the ms would be much stronger with the in vivo imaging of pollen germination on stigmatic papillae (i) GEF8/9 in wt, (ii) gef8/9 double mutant. This would bring crucial data about the role of the GEF polar domain and its functional relation to pollination.
4. The phylogeny presented in Fig S1 is only rudimental and not very interesting. Given the author's results, I would love to see if GEF8/9 orthologs also exist in species with defined pollen apertures (where establishing a dynamic site makes little sense). The authors touch on this (L409-411), but it would deserve better analysis and discussion.
5. I am not entirely convinced by the authors' interpretation of rather strange S518 mutation data. Could S518A mutation affect overall GEF8 structure/stability?
6. Although the authors cannot observe the localisation of ROPs in the plasma membrane, they see the apparent accumulation of active ROP marker CRIB4 there - implying that ROPs must localise to the pollen PM at the germination site. This discrepancy should be solved or at least discussed more.
7. Given that calcium oscillates very rapidly in pollen and pollen tubes (with frequency ~6-20s), the profound, long-term changes in calcium levels reported by the authors can hardly be referred to as oscillations. The phenomenon observed should again be analysed using a bigger sample.

Minor points

1. In Fig 1F, GEF12 also seems to be polarly localised to the future site.
2. It is difficult to make any assumptions based on the AlphaFold2 predictions without showing their confidence assessments (e.g., PAE plots). The authors state this themselves in the discussion (L. 447-449).
3. On one hand the authors repeatedly state that pollen GEFs do act in a redundant manner (and provide some evidence for it), on the other hand the absence of an in vivo phenotype for single and double knockout lines and only mild phenotype for a triple ko line does suggest a level of redundancy. This should be rephrased.

Significance (Required)):

General assessment:

I believe that both strenghts and limitations are evident form the list above. I feel this a study with great potential, which can be improved by textual ammendments and by several additional experiments that do not require the generation of new genetic material.

Advance:

This ms builds on the results obtained previously by the lead author and does advance the knowledge of the field of plant cell polarity substantially.

Audience:

The ms is targetted for the basic research audience, particularly for plant scientists.

Expertise of the reviewer:

Pollen biology, membrane trafficking, phylogenetic analyses, protein biochemistry.
