# Supplementary material for: Polarized subcellular activation of Rho proteins by specific ROPGEFs drives pollen germination in Arabidopsis thaliana
Source: PLoS Biol. 2025 Apr 21;23(4):e3003139. doi: 10.1371/journal.pbio.3003139 (PMC12043234; doi:10.1371/journal.pbio.3003139)
Supplement: Attachment — Submitted filename: Response to Reviewer_Bouatta et al_PBIOLOGY-D-24-01976R1.docx [file pbio.3003139.s033.docx]

**Manuscript number:** PBIOLOGY-D-24-01976R1

**Corresponding author(s):** Philipp, Denninger

## 1. General response

We thank all three reviewers for the constructive and well-justified comments and suggestions. For this revised version, we generated multiple new lines and included them in this manuscript, along with multiple new quantifications that were suggested by the reviewers. With all this included in this revised version, we hope that we addressed all comments and implemented all changes to the satisfaction of the reviewers. We already responded to the reviewer's comments in the revision plan earlier. This additional response states which experiments were added to the revised version. However, we also used some original answers if they did not change or only addressed questions of the reviewers. Together, we appreciate the reviewer's comments, as they significantly improve our revised manuscript.

## 2. Response to individual reviews

**Response to Reviewer #1:**

Major comments:

1. Only one GEF11 mutant line, gef11-t1, was analyzed for germination ratio. It is presumptuous to conclude that GEF11 has no function in the pollen germination of Arabidopsis thaliana (line 241- line 242).

We generated an additional *gef11* single mutant allele for this revised manuscript using CRISPR/Cas9, as no other suitable lines were available. Moreover, we generated higher-order mutants (*gef8;11, gef9;11, gef9;11;12, gef8;9;11*) by crossing or CRISPR-Cas9 and included them in this manuscript (Figure 2). This showed that *gef11* mutants have indeed no negative effect on pollen germination efficiency. In contrast, these new results indicate an opposite function of GEF11, as we discussed in the revised version.

(See also response to Reviewer 2)

Minor comments:

1. In Figure 2A, pollen germination ratio was not provided for the single mutants gef8-c△3 and gef9-c△2.

This is due to the generation process of the CRISPR/Cas9 alleles. These alleles were generated by a construct mutating both genes simultaneously; thus, these mutants are unavailable as single mutant lines. Instead of separating these alleles by outcrossing, we included additional single mutant alleles for both GEFs with a similar deletion. As all these CRISPR/Cas9 mutants have a complete deletion of the GEF-ORF, we are sure about the loss of the according GEF function. Additional alleles account for possible unspecific effects. However, to eliminate uncertainties, we clarified this in the M&M section of the revised version.

2. In Figure 3D, the subcellular localization of GEF12GEF8C is fuzzy. Better imaging is needed.

We included images of better quality in the revised version.

3. In Figure 3E, it is intriguing that both GEF8-S518A and GEF8-S518D are not associated with the PM in germinating pollen grains. Does it mean that phosphorylation at S518 is not relevant to polar distribution of GEF8?

We included a more quantitative analysis of our results (as suggested by Reviewer 3). This showed that our original interpretation of the data did not include all the information. The quantification data of this revised manuscript shows that S518A and S518D behave differently regarding their localisation. While S518A lost polarity, S518D still polarises, but to a lesser degree. It still does not fully explain how this phosphorylation site affects GEF localisation, but our interpretation is that this site needs to be in a state in which it can be phosphorylated for proper RLK interaction. Interestingly, both mutants are functional to the same degree, questioning the previous hypothesis that this phosphorylation site is required for GEF activation.

4. T-DNA insertion lines, gef11-t1 and gef12-t1, need to be verified by PCRs in Figure S3D.

This is now included in Supplemental Figure 3.

**Response to Reviewer #2:**

Major comments:

1. In line 241, the authors conclude that GEF11 has no function in pollen germination. However, it is likely that GEF11 also plays a redundant role as GEF12 does. I recommend the authors check the phenotypes of gef11,gef12 double mutant and gef8,gef9,gef11 triple mutant to confirm that GEF11 has indeed no function. Otherwise, this conclusion should be better rephrased.

We generated an additional *gef11* allele, *gef8;11, gef9;11* double mutants, and *gef9;11;12, gef8;9;11* triple mutants to address the function of *GEF11* in more detail. These results are included in the revised Figure 2. All these mutants showed no negative effect of *GEF11* on pollen germination efficiency.

(See also response to Reviewer 1)

2. Although GEF12 is in the cytosol, the strong pollen germination defects in gef8,gef9,gef12 triple mutants do indicate a critical role of GEF12. Is it possible that GEFs could function in the cytosol? The authors can test this possibility by examining the rescuing ability of several constructs that express, for example, GEF12, GEF12(+GEF8C), GEF8(SA), or GEF8(SD) in gef8. The authors may not perform all of these rescue experiments, but some of the mentioned lines are already in hands. They could readily check the phenotypes.

We have generated all suggested constructs (GEF12, GEF12^GEF8C^, GEF8^S518A^, GEF8^S518D^, and GEF8^ΔC^) in the *gef8* background to address this. These results are included in Figure 3. As discussed in the revised manuscript, it seems that cytosolic GEF8 is not functional or that the C-terminus of GEF8 has more functions than just polarising GEFs. We also show that cytosolic GEF12 can partially rescue the loss of gef8. However, as pointed out in the revised manuscript, even distribution in the cell without polarisation does not mean that such a protein is not present at the plasma membrane. Even without accumulation, there might be sufficient protein close to the membrane to fulfil a function. We hypothesise that the polarisation renders the process more efficient and stable to ensure fast and robust germination, which provides an advantage in fertilisation.

3. The authors conclude that the C-terminus of GEF8 and GEF9 is necessary and sufficient for membrane localization because GEF8/9C can target GEF12 PRONE domain to the membrane. It is intriguing whether the C-terminus alone could confer membrane targeting ability. Currently, it is not fully understood how GEFs localize to the membrane. Examining the localization of GEF8/9C itself would help clarify this and improve our understanding of GEF regulation. Alternatively, the authors may discuss evidence that supports or disagrees with this possibility.

We generated a mCit-GEF8^C-Term^ construct and checked for polarisation. However, the C-terminus alone is not sufficient to polarise and requires the PRONE context. These results are described in the revised manuscript and in Supplemental Figure 6.

Minor comments:

1. The N- and C-terminus of GEF8 are predicted to inhibit complex formation. How is the prediction performed? Do the authors use monomer prediction or multimer prediction? Alphafold2 has a low accuracy in predicting non-conserved regions. How confident are the predicted inhibitory contacts?

We included the additional Supplemental Figure S5 to address these questions and provide more data on the structure predictions. We also added sections to the revised manuscript describing the model in more detail, and we changed our interpretation of the structures due to the uncertainty of the prediction in the terminal region, as suggested by reviewer 3.

(See also response to Reviewer 2)

2. Localization of ROPs and calcium reporter in Figure 4 appears to be variable. It would help clarify the specific effects on each reporter if the authors present these data more quantitatively.

In the revised manuscript, we include quantified data to better describe our observations. This changed some of our interpretations, which were altered accordingly in the manuscript (See Figure 4). However, our original conclusion that GEF8 influences ROP activity and Ca2+ levels could be confirmed.

(see also Response to Reviewer #3).

**Response to Reviewer #3:**

Major points:

1. One of my major points is that the manuscript is now mainly based on the observations of individual pollen grains. These are then subjected to well-performed image analysis approaches but still represent somewhat anecdotal evidence (Fig 1A, B, Fig 3C-E, etc). The analysis and (numerical) presentation of a more robust data sample (which I presume the authors have acquired) would strengthen the ms considerably. This goes beyond the Figs - e.g. in l. 164-165 authors state rather vaguely, "we observed that mCit-GEF8 and mCit-GEF9 accumulated at a defined region in the cell periphery, which strongly correlated with the future germination site." Here, I would appreciate the data showing the actual correlation, if every germinated pollen grain displays GEF8/9 accumulation, whether there is a population of pollen grains showing the GEF8/9 transient but not germinating, etc...

We thank the Reviewer for this comment. We based our initial results on more than just single observations, as the reviewer correctly presumed. However, we fully agree with the reviewer that our initial version of the manuscript was not quantitative and precise enough. Thus, we revisited our data and quantified multiple parameters to provide more information and details on the polarisation properties of each construct. We included new quantifications in Figure 1, Figure 3, and Figure 4, together with supplemental Figures to provide more information on our data. Additionally, we adjusted the revised manuscript, describing our quantifications and additionally phrased our descriptions in a more specific way. With the suggested quantification, we were also able to draw some new conclusions that significantly improved our manuscript.

2. Where the authors analyse multiple cells, we are still missing some info - e.g. it is not stated what the error bars in Fig 1C, D represents (SD, SEM, CI?), size of the sample, etc. In any case, it is evident that there is quite substantial variability in the data, which is understandable. Maybe the authors can plot the individual profile lines along the average? Plus, GEF9 seem to have the maximum pre-germination localisation at -5 min rather than -9 min.

Also, in this case, the reviewer raises a good point. We followed the suggestion and showed the single traces of the measurements to give a better impression of the variability. This more quantitative visualisation also allowed us to specify our description of the localisation. Additionally, we included numbers for sample size and significance levels for all quantitative data and showed plots with all data points to provide a transparent view of our data.

3. I know it is very challenging, but the ms would be much stronger with the in vivo imaging of pollen germination on stigmatic papillae (i) GEF8/9 in wt, (ii) gef8/9 double mutant. This would bring crucial data about the role of the GEF polar domain and its functional relation to pollination.

As stated in the revision plan, this would indeed be very informative. However, with our resources and the available lines, we were not able to get reliable imaging of the events. Establishing this new technique would exceed the scope of this project, and even though we agree that this could provide further information in the future, we are convinced that our presented results in this revised manuscript are novel, convincing, and of importance to the community.

4. The phylogeny presented in Fig S1 is only rudimental and not very interesting. Given the author's results, I would love to see if GEF8/9 orthologs also exist in species with defined pollen apertures (where establishing a dynamic site makes little sense). The authors touch on this (L409-411), but it would deserve better analysis and discussion.

We agree with the reviewer that studying GEF function/accumulation in species with aperture-dependent germination would be interesting. However, we can not conclude functional orthologs in other species based on phylogeny. Such phylogenetic analyses were done, for example, by Kim et al. (BMC Plant Biology, 2020, doi: 10.1186/s12870-020-2298-5). The issue is that all Arabidopsis pollen-expressed GEFs form a closed phylogenetic group without allowing the interpretation of which rice homolog is the functional ortholog of the respective Arabidopsis GEF (this is the same for maize). Thus, such phylogenetic analyses are not conclusive, and they would require experimental data to prove orthology. However, we agree that this point can be interpreted and discussed better. In this revised version, we described the phylogeny in more detail and also the difficulty of finding functional orthologues. Additionally, we discussed the question of why species that germinate at the aperture also diversified GEFs and if they need functional orthologues for the early accumulating GEFs.

5. I am not entirely convinced by the authors' interpretation of rather strange S518 mutation data. Could S518A mutation affect overall GEF8 structure/stability?

We included more data on these lines, along with the quantification mentioned earlier, and we can now describe in more detail how these lines behave. However, we still do not fully understand the function of this phosphorylation site. It would require multiple new lines and experiments to fully understand how this phosphorylation site affects localisation, activity and interaction with other proteins. This would exceed the scope of this manuscript. In this revised manuscript, we include the novel data and quantifications and also discuss our new conclusions and possible explanations. One explanation discussed, as the reviewer suggests, is that our mutations affect the GEF8 structure in different ways, leading to the observed different outputs.

6. Although the authors cannot observe the localisation of ROPs in the plasma membrane, they see the apparent accumulation of active ROP marker CRIB4 there - implying that ROPs must localise to the pollen PM at the germination site. This discrepancy should be solved or at least discussed more.

As stated in the response to Reviewer #2 on GEF12 localisation (Major comment #2), there is not necessarily a discrepancy in our initial statement. We can not observe any accumulation of ROPs at the pollen germination site. However, this does not exclude their presence at the plasma membrane of this location. Normal confocal microscopy does not have sufficient resolution to determine if proteins are directly associated with the plasma membrane. Such conclusions can only be drawn if specific accumulations are observed. Even without accumulation and an even distribution in the cytosol, ROPs should also be present close to the plasma membrane, which could be sufficient to activate ROP signalling. We included several statements in the results and discussion section to clarify this and to avoid the impression that ROPs or GEF12 are excluded from the site just because we do not see them accumulating.

7. Given that calcium oscillates very rapidly in pollen and pollen tubes (with frequency ~6-20s), the profound, long-term changes in calcium levels reported by the authors can hardly be referred to as oscillations. The phenomenon observed should again be analysed using a bigger sample.

We changed our wording to clearly distinguish our observed Ca^2+^ elevations from the known oscillations, using “elevations” or “Ca^2+^ level”. Additionally, we analysed and quantified more observations, which also slightly changed our initial interpretation of the observations but did not change our overall conclusion that GEF8 regulated Ca^2+^ levels. We provided these new quantitative results in Figure 4.

Minor points:

1. In Fig 1F, GEF12 also seems to be polarly localised to the future site.

As seen in the quantification of the revised Figure 1, GEF12 rarely accumulated with low polarity at the pollen germination site. The initial example showed such an exception but also showed a clear difference in polarity levels of GEF8 and GEF12. However, in the revised version of Figure 1, we selected a more representative example without GEF12 accumulation.

2. It is difficult to make any assumptions based on the AlphaFold2 predictions without showing their confidence assessments (e.g., PAE plots). The authors state this themselves in the discussion (L. 447-449).

We now included the model of GEF8 with pLLDT visualisation for all five models provided by the prediction, including PAE plots in Supplemental Figure 5. This gives a better impression of the confidence in the structure of the terminal regions, which is low and likely disordered. As the reviewer states, it is hardly possible to draw any conclusions from this. We changed all statements about the structure of the terminal regions in the revised manuscript to ensure that the provided structure prediction only allows speculations and is not confident enough to draw any conclusions.

3. On one hand the authors repeatedly state that pollen GEFs do act in a redundant manner (and provide some evidence for it), on the other hand the absence of an in vivo phenotype for single and double knockout lines and only mild phenotype for a triple ko line does suggest a level of redundancy. This should be rephrased.

We do find genetic redundancy as we observe increasing phenotypic strength in higher-order mutants. However, this only applies to *GEF8, GEF9,* and *GEF12*, not GEF11, as *gef11* mutants do not show such redundant behaviour. Additionally, we find that GEF12 is not redundant to GEF8, GEF9, or GEF11 concerning the cell's biological function, as it is localised differently. Therefore, a statement of redundancy is not as easy, as this affects multiple layers of functions and is apparently different for each group of GEFs. In the revised manuscript, we changed and extended our statements on redundancy between GEFs to clarify our conclusions and interpretations.

## 3. Original Reviews:

**Reviewer #1 (Evidence, reproducibility and clarity (Required)):**

In the manuscript, the Denninger group reported the identification of ROPGEF8/9 as the key ROPGEFs for ROP activation during pollen germination, a process of polarity establishment.
By examining the subcellular localization of pollen-expressed/enriched GEFs using their own promoter and fluorescence protein fusions, the authors convincingly showed the spatiotemporal distribution of GEF8 and GEF9 during pollen germination. By characterizing pollen germination of gef mutants, the authors demonstrated that GEF8 and GEF9 are critical for the process, with GEF12 playing a redundant role likely as a compensation response. The authors further showed that C-termini of GEF8/9, previously demonstrated as an inhibitory domain for GDP-GTP exchange, was critical for the polar distribution. The C-termini of GEFs interact with PRK. The authors reported that the phosphorylation of GEFs at C-termini was critical for their polar distribution. By examining the dynamic localization of active ROP biosensor CRIBRIC4, the authors demonstrated that GEF8/9 were critical for polar distribution of active ROPs at future germination sites. By introducing a calcium biosensor, the authors showed that calcium gradient, a key downstream process of ROP signaling, was compromised by functional loss of GEF8/9 during pollen germination.

**major comments**

4. Only one GEF11 mutant line, gef11-t1, was analyzed for germination ratio. It is presumptuous to conclude that GEF11 has no function in the pollen germination of Arabidopsis thaliana (line 241- line 242).

**minor comments**

1. In Figure 2A, pollen germination ratio was not provided for the single mutants gef8-c△3 and gef9-c△2.
5. In Figure 3D, the subcellular localization of GEF12GEF8C is fuzzy. Better imaging is needed.
6. In Figure 3E, it is intriguing that both GEF8-S518A and GEF8-S518D are not associated with the PM in germinating pollen grains. Does it mean that phosphorylation at S518 is not relevant to polar distribution of GEF8?
7. T-DNA insertion lines, gef11-t1 and gef12-t1, need to be verified by PCRs in Figure S3D.

**Significance (Required)):**

The identification of ROPGEF8/9 as the key ROPGEFs for ROP activation during pollen germination is a step forward in understanding ROP signaling. Useful but not unexpected. Pollen germination is a process of polarity establishment, similar to root hair initiation. Compared to pollen tube growth and root hair growth, processes of polarity maintenance, the role of ROP signaling was less clear. Recently, Xiang et al. (2023, Plant Physiol) reported an essential role of ROP1/3/5 and their downstream components BDR8/9 in pollen germination. Consistently with polar ROP activation, Ca2+ and post-Golgi secretion were polar. The current work is one step ahead, showing GEF8/9 as the upstream GEFs for this process, comparable to GEF3/4 during root hair initiation (Denninger et al., 2019, Curr Biol). The identification of the C-terminal phosphorylate site in GEF8/9 is informative. It was reported previously that PRKs interact with the C-termini of GEFs to release their auto-inhibition (Gu et al., 2006, Plant Cell; Zhang and McCormick, et al., 2007, Proc Nat Acad Sci USA, Zhao et al., 2013, J Exp Bot) and PRKs were reported to phosphorylate GEF (Chang et al., 2013, Mol Plant). Thus, results reported in the current work indicate that phosphorylation of GEFs likely by PRKs is a critical step for the establishment of polarity domain for pollen germination. From this perspective, it would be more mechanistically sound to investigate the role of PRKs in spatiotemporal polarization of GEFs during pollen germination.Researchers working on cell signaling and cell morphogenesis in plants will be interested. My lab works on cell morphogenesis and ROP signaling. This manuscript exactly falls within the expertise of my field.

**Reviewer #2 (Evidence, reproducibility and clarity (Required)):**

In this study, Bouatta et al. report the function of RopGEFs in pollen germination. The authors analyzed all of the five RopGEFs, namely RopGEF8/9/11/12/13, that have been shown to be expressed in mature pollen tubes, and found that only GEF8/9/11/12 are detectable. In addition, GEF8 and GEF9 localize to germination sites, while GEF11 and GEF12 are cytosolic. Through a series of phenotype analyses and live-cell imaging, the authors show that GEF8, GEF9, and GEF12 are required for pollen germination while GEF11 is not. The authors also provide evidence that GEF8 and GEF9 are targeted to the membrane via the C-terminus, where they activate ROPs and calcium signaling.

**Major comments:**

1. In line 241, the authors conclude that GEF11 has no function in pollen germination. However, it is likely that GEF11 also plays a redundant role as GEF12 does. I recommend the authors check the phenotypes of gef11,gef12 double mutant and gef8,gef9,gef11 triple mutant to confirm that GEF11 has indeed no function. Otherwise, this conclusion should be better rephrased.
2. Although GEF12 is in the cytosol, the strong pollen germination defects in gef8,gef9,gef12 triple mutants do indicate a critical role of GEF12. Is it possible that GEFs could function in the cytosol? The authors can test this possibility by examining the rescuing ability of several constructs that express, for example, GEF12, GEF12(+GEF8C), GEF8(SA), or GEF8(SD) in gef8. The authors may not perform all of these rescue experiments, but some of the mentioned lines are already in hands. They could readily check the phenotypes.
3. The authors conclude that the C-terminus of GEF8 and GEF9 is necessary and sufficient for membrane localization because GEF8/9C can target GEF12 PRONE domain to the membrane. It is intriguing whether the C-terminus alone could confer membrane targeting ability. Currently, it is not fully understood how GEFs localize to the membrane. Examining the localization of GEF8/9C itself would help clarify this and improve our understanding of GEF regulation. Alternatively, the authors may discuss evidence that supports or disagrees with this possibility.

**Minor comments:**

1. The N- and C-terminus of GEF8 are predicted to inhibit complex formation. How is the prediction performed? Do the authors use monomer prediction or multimer prediction? Alphafold2 has a low accuracy in predicting non-conserved regions. How confident are the predicted inhibitory contacts?
2. Localization of ROPs and calcium reporter in Figure 4 appears to be variable. It would help clarify the specific effects on each reporter if the authors present these data more quantitatively.

**Advance:** ROP GTPases and RopGEFs are critical regulators of cell polarity, but how they initiate polarity remains unclear. This study uses pollen germination as a model to address this question. It systematically analyzed all pollen-specific GEFs and found that GEF8 and GEF9 are critical regulators of pollen germination and polarity initiation. Importantly, GEF8 and GEF9 undergo biphasic accumulation, suggesting polarity is established through a transient exploration phase. This study provides a comprehensive view of the functions of GEFs in polarity initiation, which will be of interest not only to readers who work on pollen germination and growth but also to those who study cell polarity and morphogenesis in general. In my view, the most novel part of this study is that GEFs play overlapping but non-identical roles in polarity establishment and undergo transient accumulation during the polarity initiation process.

**Limitations:** This study shows that GEFs use the C-terminus for membrane targeting and GEFs can activate ROPs and calcium signaling during pollen germination. These mechanisms could be largely inferred from previous studies in mature pollen tubes or others. Advancements in the regulation of GEF such as how the C-terminus mediates GEF localization, e.g. whether through direct interaction with the PRONE domain in a phosphorylation-dependent manner, would further increase the novelty of this work.

**Reviewer #3 (Evidence, reproducibility and clarity (Required)):**

This manuscript investigates the role of PRONE ROP GEFS in germinating Arabidopsis pollen. Given that the molecular mechanisms underlying cellular polarisation in pollen germinating pollen grains are still largely unknown (as opposed to the tip growth of elongating pollen tubes), this manuscript deals with an important topic. Moreover, it builds on the excellent previous research from the lead author, which uncovered ROP GEFs as principal polarisation players during root hair initiation. Here, the authors found that out of five pollen-expressed GEFS, two (GEF8 and 9) mark a future germination site with remarkable spatiotemporal dynamics. Using the genetic tools, GEF8 and 9 were shown to be important for pollen germination in vitro and participate in germination in vivo. Generally, this is an exciting topic, and I quite enjoyed reading the manuscript. However, there are several aspects of the work, which - when addressed - would significantly improve the overall message presented by the authors.

**Major points**

1. One of my major points is that the manuscript is now mainly based on the observations of individual pollen grains. These are then subjected to well-performed image analysis approaches but still represent somewhat anecdotal evidence (Fig 1A, B, Fig 3C-E, etc). The analysis and (numerical) presentation of a more robust data sample (which I presume the authors have acquired) would strengthen the ms considerably. This goes beyond the Figs - e.g. in l. 164-165 authors state rather vaguely, "we observed that mCit-GEF8 and mCit-GEF9 accumulated at a defined region in the cell periphery, which strongly correlated with the future germination site." Here, I would appreciate the data showing the actual correlation, if every germinated pollen grain displays GEF8/9 accumulation, whether there is a population of pollen grains showing the GEF8/9 transient but not germinating, etc...
2. Where the authors analyse multiple cells, we are still missing some info - e.g. it is not stated what the error bars in Fig 1C, D represents (SD, SEM, CI?), size of the sample, etc. In any case, it is evident that there is quite substantial variability in the data, which is understandable. Maybe the authors can plot the individual profile lines along the average? Plus, GEF9 seem to have the maximum pre-germination localisation at -5 min rather than -9 min.
3. I know it is very challenging, but the ms would be much stronger with the in vivo imaging of pollen germination on stigmatic papillae (i) GEF8/9 in wt, (ii) gef8/9 double mutant. This would bring crucial data about the role of the GEF polar domain and its functional relation to pollination.
4. The phylogeny presented in Fig S1 is only rudimental and not very interesting. Given the author's results, I would love to see if GEF8/9 orthologs also exist in species with defined pollen apertures (where establishing a dynamic site makes little sense). The authors touch on this (L409-411), but it would deserve better analysis and discussion.
5. I am not entirely convinced by the authors' interpretation of rather strange S518 mutation data. Could S518A mutation affect overall GEF8 structure/stability?
6. Although the authors cannot observe the localisation of ROPs in the plasma membrane, they see the apparent accumulation of active ROP marker CRIB4 there - implying that ROPs must localise to the pollen PM at the germination site. This discrepancy should be solved or at least discussed more.
7. Given that calcium oscillates very rapidly in pollen and pollen tubes (with frequency ~6-20s), the profound, long-term changes in calcium levels reported by the authors can hardly be referred to as oscillations. The phenomenon observed should again be analysed using a bigger sample.

**Minor points**

1. In Fig 1F, GEF12 also seems to be polarly localised to the future site.
2. It is difficult to make any assumptions based on the AlphaFold2 predictions without showing their confidence assessments (e.g., PAE plots). The authors state this themselves in the discussion (L. 447-449).
3. On one hand the authors repeatedly state that pollen GEFs do act in a redundant manner (and provide some evidence for it), on the other hand the absence of an in vivo phenotype for single and double knockout lines and only mild phenotype for a triple ko line does suggest a level of redundancy. This should be rephrased.

**Significance (Required)):**

General assessment:

I believe that both strenghts and limitations are evident form the list above. I feel this a study with great potential, which can be improved by textual ammendments and by several additional experiments that do not require the generation of new genetic material.

Advance:

This ms builds on the results obtained previously by the lead author and does advance the knowledge of the field of plant cell polarity substantially.

Audience:

The ms is targetted for the basic research audience, particularly for plant scientists.

Expertise of the reviewer:

Pollen biology, membrane trafficking, phylogenetic analyses, protein biochemistry.
